# Temporal transcriptomic atlas reveals sequential engagement of classical and emerging regulated cell death pathways during PRRSV infection

**DOI:** 10.3389/fvets.2025.1737270

**Published:** 2026-01-06

**Authors:** Shinuo Cao, Li Zhang, Rui Zhu, Nannan Nie, Zhi Wu, Shanyuan Zhu

**Affiliations:** Swine Infectious Diseases Division, Jiangsu Key Laboratory for High-Tech Research and Development of Veterinary Biopharmaceuticals, Engineering Technology Research Center for Modern Animal Science and Novel Veterinary Pharmaceutic Development, Jiangsu Agri-animal Husbandry Vocational College, Taizhou, Jiangsu, China

**Keywords:** apoptosis, cuproptosis, ferroptosis, immunogenic cell death, PRRSV, pyroptosis, regulated cell death, transcriptomics

## Abstract

Porcine reproductive and respiratory syndrome virus (PRRSV) causes devastating economic losses through complex immunopathology, yet the molecular mechanisms orchestrating host cell fate remain elusive. Here we conducted temporal transcriptomic profiling of PRRSV-infected MARC-145 cells at 0, 12, 24, 36, 48, and 72 h post-infection, revealing the dynamic and temporally coordinated regulation of distinct regulated cell death (RCD) pathways. We discovered that PRRSV employs a sophisticated temporal strategy. The ferroptosis-related modules responsible for regulating lipid peroxidation (including ACSL4, LPCAT3, ALOX15, NOX1, and NCOA4) were largely downregulated, whereas cytoprotective elements (such as HSPB1, SLC40A1, HSPA5, GCLC, and later SLC7A11) were upregulated. Pathway scores remained negative up to approximately 48 h.p.i., gradually approaching neutrality by 72 h.p.i., suggesting that viral mechanisms may inhibit iron-dependent lethal lipid damage. Notably, we identified novel engagement of emerging RCD modalities-cuproptosis showed biphasic regulation with late activation through ATP7A/B suppression, while disulfidptosis signatures peaked at 36 h.p.i. via SLC3A2/SLC7A11 induction. Immunogenic cell death signatures persisted throughout infection with sustained HMGB1 elevation. These findings reveal PRRSV extensively modulates cell death transcriptional programs through temporally coordinated strategies: early inflammatory priming with lytic pathway suppression (12–24 h.p.i.), mid-phase ER stress and organellar remodeling (24–48 h.p.i.), and late metabolic and pH vulnerabilities (48–72 h.p.i.). These transcriptional profiles, pending functional validation, suggest how the virus may balance replication permissiveness with controlled cytopathology. These insights into temporal staging of RCD pathway modulation guide targeted interventions timed to specific infection phases to reduce disease while enhancing antiviral immunity.

## 1 Introduction

Porcine reproductive and respiratory syndrome virus (PRRSV) is an enveloped, positive-sense, single-stranded RNA virus classified within the genus Betaarterivirus (1, 2). It is one of the most economically detrimental pathogens affecting the global swine industry (3, 4). Highly pathogenic PRRSV (HP-PRRSV) lineages, characterized by a distinctive discontinuous 30 amino acid deletion in the nsp2 protein, have been responsible for severe outbreaks, causing unusually severe systemic disease (5–7). HP-PRRSV infection in pigs leads to high fever, severe lethargy, difficulty breathing, “blue ear” cyanosis, skin spots, reproductive issues in breeding herds, and increased mortality, especially in young pigs (8). The virus primarily affects the lungs and lymphoid organs, with significant replication in the lungs, tonsils, lymph nodes, and spleen, and is also found in the liver and kidneys. Pathologically, it causes diffuse interstitial pneumonia with alveolar thickening, type II pneumocyte proliferation, and macrophage infiltration, often accompanied by pulmonary edema and bacterial bronchopneumonia (9). Lymphoid tissues show severe depletion and necrosis with histiocytic infiltration, indicating strong immunosuppression. Vasculitis and hemorrhages point to endothelial damage and systemic inflammation. HP-PRRSV replicates extensively in macrophages, suppresses antiviral responses, alters cytokine profiles, and causes apoptosis and pyroptosis in immune cells, leading to lymphopenia and poor antigen presentation (10). These observations of virus-induced apoptosis and pyroptosis in immune cells underscore the central importance of regulated cell death pathways in PRRSV pathogenesis, necessitating a comprehensive examination of the molecular mechanisms governing these cellular responses.

Regulated cell death (RCD) constitutes a fundamental biological process, characterized by intricately controlled signaling pathways and molecularly defined effector mechanisms that coordinate cellular disassembly in response to physiological or pathological stimuli (11, 12). RCD encompasses precise molecular machinery that can be modulated through genetic interventions (13). This distinction holds significant implications for understanding disease pathogenesis and developing therapeutic strategies, as RCD is pivotal in maintaining organismal homeostasis, eliminating damaged or infected cells, and regulating immune responses (13, 14). The classification of RCD has evolved considerably from its initial morphological categorization to a sophisticated molecular taxonomy (15). The current nomenclature, as recommended by the Nomenclature Committee on Cell Death (NCCD), acknowledges multiple distinct subroutines, including apoptosis, necroptosis, pyroptosis, ferroptosis, parthanatos, entosis, and NETosis (16). Lysosome-dependent and autophagy-dependent cell death are processes linked to regulated cell death (RCD) but are not considered independent RCD pathways (17, 18). Viral infections, such as those caused by PRRSV, highlight how RCD intersects with host defenses and viral strategies (19). Viruses can manipulate host cell death to either promote viral spread or inhibit RCD to prolong replication (20, 21). This virus induces significant cell death in immune cells and modulates inflammation, illustrating the complex host-pathogen dynamics (22, 23). The virus causes apoptosis in infected macrophages via caspase pathways and can also trigger pyroptosis through inflammasome activation, leading to lung injury and higher risk of secondary bacterial infections (24). By altering RCD pathways, the virus affects the immune response, influencing whether the host develops effective immunity or suffers immune dysregulation (25). Understanding these mechanisms is crucial for identifying targets to reduce tissue damage while maintaining protective immunity. PRRSV provides a valuable model system for elucidating the intricate interplay of these RCD pathways in viral pathogenesis.

The cellular response to PRRSV infection encompasses various forms of regulated cell death, with the specific mechanisms and their temporal and contextual roles remaining active areas of research (26). Early investigations indicated that the viral envelope protein GP5 may play a role in facilitating the induction of apoptosis. However, later research has uncovered a more complex situation (27). Apoptosis is the most thoroughly documented form of cell death associated with PRRSV infection, characterized by extensive activation of caspase-dependent pathways in infected macrophages and lymphoid tissues (28). Although some studies have indicated the activation of receptor-interacting protein kinase pathways, implying a potential role for necroptosis, the direct contribution of this pathway to PRRSV pathogenesis warrants further exploration (29). The virus appears to employ strategies to modulate apoptosis, including the upregulation of cellular factors such as heat shock protein 70 (30). Nevertheless, the role of activating transcription factor 3 is context-dependent and not universally anti-apoptotic. Intrinsic apoptosis is not entirely blocked, as apoptosis is commonly seen in infected tissues (31). Recent studies show that pyroptosis plays a key role in PRRSV pathogenesis by activating inflammasomes, leading to gasdermin D-mediated pore formation (26). This releases proinflammatory cytokines and damage signals, increasing inflammation and tissue damage. Additionally, oxidative stress and lipid peroxidation from PRRSV replication suggest ferroptosis might be involved, though evidence is still emerging (19). To elucidate the temporal regulation of cell death pathways during PRRSV infection, we employed comprehensive transcriptomic profiling at defined intervals (0, 12, 24, 36, 48, and 72 h post-infection). This systematic temporal analysis enables identification of phase-specific transcriptional programs governing apoptotic, necroptotic, pyroptotic, and ferroptotic machinery. Through high-resolution mapping of gene expression dynamics, this study aims to delineate the sequential activation and cross-regulation of cell death pathways, identify critical regulatory nodes amenable to therapeutic intervention, and establish the molecular basis for PRRSV-induced cytopathology. These findings will provide fundamental insights into viral manipulation of host cell fate decisions and inform rational design of targeted interventions that mitigate tissue damage while preserving antiviral immunity.

## 2 Materials and methods

### 2.1 Cell culture and virus infection

MARC-145 cells (African green monkey kidney epithelial cells) were cultured in Dulbecco’s Modified Eagle’s Medium (DMEM; Gibco, Thermo Fisher Scientific, Waltham, MA, United States) supplemented with 10% fetal bovine serum (FBS; Gibco), 100 U/mL penicillin, and 100 μg/mL streptomycin (Sigma-Aldrich, St. Louis, MO, United States). Cells were incubated at 37 °C in a humidified 5% CO₂ atmosphere. The PRRSV-2 modified live vaccine strain R98 was propagated in MARC-145 cells. Viral titer was quantified by 50% tissue culture infectious dose (TCID_50_) assay in MARC-145 cells. For infection, MARC-145 cells were seeded in 6-well plates at 5 × 10^5^ cells per well and grown to 80% confluence. Cells were infected with PRRSV at a multiplicity of infection (MOI) of 10, and samples were collected at 0, 12, 24, 36, 48, and 72 h post-infection (h.p.i.). Mock-infected controls received an equivalent volume of virus-free medium. All experiments were conducted in biological triplicate.

### 2.2 Indirect immunofluorescence assay

After infection with PRRSV for identified hours, MARC-145 were fixed by 4% paraformaldehyde (PFA), permeabilized by 0.3% Triton X-100, and then blocked with 5% bovine serum albumin (BSA). The cells were incubated for 1 h with positive sera of PRRSV (1:300) at room temperature. Unbound antibody was washed away, and then an optimum dilution (1:500) of Fluorescein (FITC)-conjugated AffiniPure Goat Anti-pig IgG (H + L) was incubated for 1 h. The cells were observed under a fluorescence microscope (Zeiss Axiovert 200).

### 2.3 RNA extraction, library construction, and sequencing

Total RNA was isolated from PRRSV-infected and uninfected MARC-145 cells with TRIzol Reagent (Invitrogen, Thermo Fisher Scientific) according to the supplier’s instructions. RNA quality was evaluated on an Agilent 2100 Bioanalyzer (Agilent Technologies, Santa Clara, CA, United States), selecting samples with RNA Integrity Number (RIN) exceeding 8.0 for further steps. RNA quantity was determined via Qubit RNA Assay Kit (Thermo Fisher Scientific). Library construction utilized 1 μg total RNA per sample. mRNA was purified using oligo (dT) beads (Dynabeads mRNA Purification Kit, Invitrogen). Purified mRNA underwent fragmentation in buffer at 94 °C for 5 min, then first-strand cDNA synthesis with random hexamers and SuperScript II (Invitrogen). Second-strand cDNA was generated using DNA Polymerase I, RNase H, and dNTPs (New England Biolabs, Ipswich, MA, United States). Double-stranded cDNA was purified by AMPure XP beads (Beckman Coulter, Brea, CA, United States), subjected to end repair, A-tailing, and adapter ligation for Illumina sequencing. Fragments of 200–300 bp were selected through agarose gel electrophoresis followed by AMPure XP purification. PCR amplification (15 cycles) employed Phusion High-Fidelity DNA Polymerase (New England Biolabs), with libraries quantified by Qubit dsDNA HS Assay Kit. Paired-end 150 bp sequencing occurred on Illumina NovaSeq 6000 (Illumina, San Diego, CA, United States) at Novogene Co., Ltd. (Beijing, China). Fastp (v0.20.1) processed raw reads by trimming adapters, discarding bases with *Q* < 20, and excluding reads below 50 bp, producing clean data for subsequent analysis.

### 2.4 Data preprocessing and differential gene expression analysis

Transcriptomic sequencing reads were initially processed and aligned to the *Sus scrofa* reference genome (e.g., Sscrofa11.1, Ensembl release 104) utilizing HISAT2 (v2.2.1). The resultant alignment files were subsequently sorted and indexed with SAMtools (v1.15). Gene expression levels were quantified from the processed reads using featureCounts (v2.0.1) within the Subread package, thereby yielding raw count matrices. For robust differential expression analysis, normalization and statistical testing were executed using the DESeq2 package (v1.34.0) implemented in R (v4.2.1). Prior to analysis, genes exhibiting negligible expression (defined as a mean count across all samples of < 10) were stringently filtered out. Differential gene expression (DEG) analysis was performed on normalized read counts using the DESeq2 package. Genes were defined as differentially expressed if they met the dual criteria of an Adjusted *p* value (FDR) < 0.05 and the absolute value of the |log2 Fold Change| ≥ 1 when comparing PRRSV-infected samples at each time point against the 0 h.p.i. control. The visualization of the DEGs was accomplished via Volcano plots constructed using the ggplot2 package (v3.3.6), distinguishing between significantly upregulated and downregulated genes. Potential batch effects were statistically mitigated by applying the ComBat function, integrated within the sva package (v3.42.0). Finally, quality control metrics, including read distribution profiles and GC content, were systematically evaluated using MultiQC (v1.11).

### 2.5 Principal component analysis

Principal component analysis (PCA) was conducted to assess sample grouping and dispersion via the prcomp function from R’s stats package. DESeq2-normalized counts underwent regularized log transformation (rlog) for variance stabilization. ggplot2 visualized the initial two principal components (PC1 and PC2), including 95% confidence ellipses to denote group distinctions. This approach enabled outlier detection and validation of biological replicate uniformity, verifying clear segregation of PRRSV-infected samples from mock controls.

### 2.6 Functional annotation and pathway enrichment analysis

Differentially expressed genes were functionally annotated utilizing an appropriate species-specific annotation package (e.g., *Sus scrofa* for porcine data). Enrichment analysis of Gene Ontology (GO) terms was subsequently executed using the clusterProfiler R package, classifying significant terms into three principal domains: Biological Process (BP), Cellular Component (CC), and Molecular Function (MF). Statistical assessment was performed employing the hypergeometric distribution test, and terms exhibiting an adjusted *p*-value (padj) less than 0.05 were designated as statistically significant. Furthermore, mapping of DEGs to the Kyoto Encyclopedia of Genes and Genomes (KEGG) pathways was conducted via clusterProfiler for pathway enrichment evaluation. The most significantly enriched GO terms and KEGG pathways were visually presented, typically through bubble plots or bar graphs, facilitated by the enrichplot package. An integrative approach synthesized the GO and KEGG findings to accentuate prominent biological mechanisms, such as those governing metabolic processes and regulated cell death.

### 2.7 Expression analysis of cell death pathways

Genes linked to multiple cell death mechanisms were assembled from public repositories. To comprehensively characterize the transcriptional modulation of RCD pathways during PRRSV infection, we curated gene sets for the distinct RCD modalities from peer-reviewed literature and specialized databases. Apoptosis-related genes were sourced from Wang et al. (32), identifying key regulators like MAP4K4 involved in phosphorylation-mediated cell fate decisions. Pyroptosis genes were derived from Xiang-Xia et al. (33), focusing on inflammation-linked markers in spinal cord injury models. Necroptosis genes were obtained from Zhang et al. (34), validated in head and neck squamous cell carcinoma. Ferroptosis regulators were extracted from FerrDb. Cuproptosis genes came from Jiang et al. (35), correlating transcriptional alterations with esophageal carcinoma malignancy. Disulfidptosis genes were drawn from Chen et al. (36), exploring disulfide stress networks. Autophagy genes were collected from the Human Autophagy Database. Lysosome-dependent cell death genes were sourced from Liu et al. (37) in colon adenocarcinoma. Parthanatos genes originated from Wu et al. (38) via multi-omics in stomach adenocarcinoma. MPT-driven necrosis genes were from RCDdb. Alkaliptosis genes were from Xiong et al. (39) in lung adenocarcinoma. Paraptosis genes were queried via GeneCards. Immunogenic cell death genes were sourced from RCDdb. These curated sets enabled overlap analysis with DEGs, revealing predominant downregulation in PRRSV-infected cells across pathways, as visualized in heatmaps (Z-score scaled).

### 2.8 Mfuzz clustering analysis

Raw transcriptome data from PRRSV-infected MARC-145 cells at 0, 12, 24, 36, 48, and 72 h.p.i. were processed to construct a standardized time-series expression matrix. Duplicate genes were removed by retaining the first occurrence per gene ID at each time point. Column headers were standardized as “ID” (gene identifier) and “h0” to “h72” (expression values). Common genes across all time points were identified via iterative intersection. Data were merged using shared gene IDs and h0 values, yielding a matrix with rows as genes and columns as time points. The expression matrix (excluding ID column) was converted to an ExpressionSet object. Genes with > 25% missing values were filtered; remaining missing values were imputed with mean values. Low-variance genes (standard deviation < 0.1) were excluded. The matrix was standardized (mean = 0, variance = 1). Clustering was performed with candidate cluster numbers (8) to select the optimal based on resolution and biological relevance. Membership degrees (0–1) were computed; values < 0 were assigned −1 to denote low affiliation.

All data generated and analyzed from this study are included in this published article. The raw RNA-Seq data has been submitted to NCBI Short Read Archive (SRA) under Bioproject PRJNA1372434 (https://www.ncbi.nlm.nih.gov/bioproject/1372434).

### 2.9 Data availability and statistical analysis

The raw RNA-seq reads have been submitted to the NCBI SRA database under BioProject accession PRJNA1137426. Unless otherwise noted, all data processing occurred in R software (version 4.2.1). GraphPad Prism (version 9.0; GraphPad Software, San Diego, CA, United States) assessed statistical significance, expressing values as mean ± SD across three replicates. Two-group differences employed unpaired *t*-tests, denoting significance as *p < 0.05, **p < 0.01, ***p < 0.001. The Benjamini-Hochberg method controlled FDR for multi-group adjustments.

## 3 Results

### 3.1 PRRSV replication kinetics and cytopathic effect

**Figure 1:**
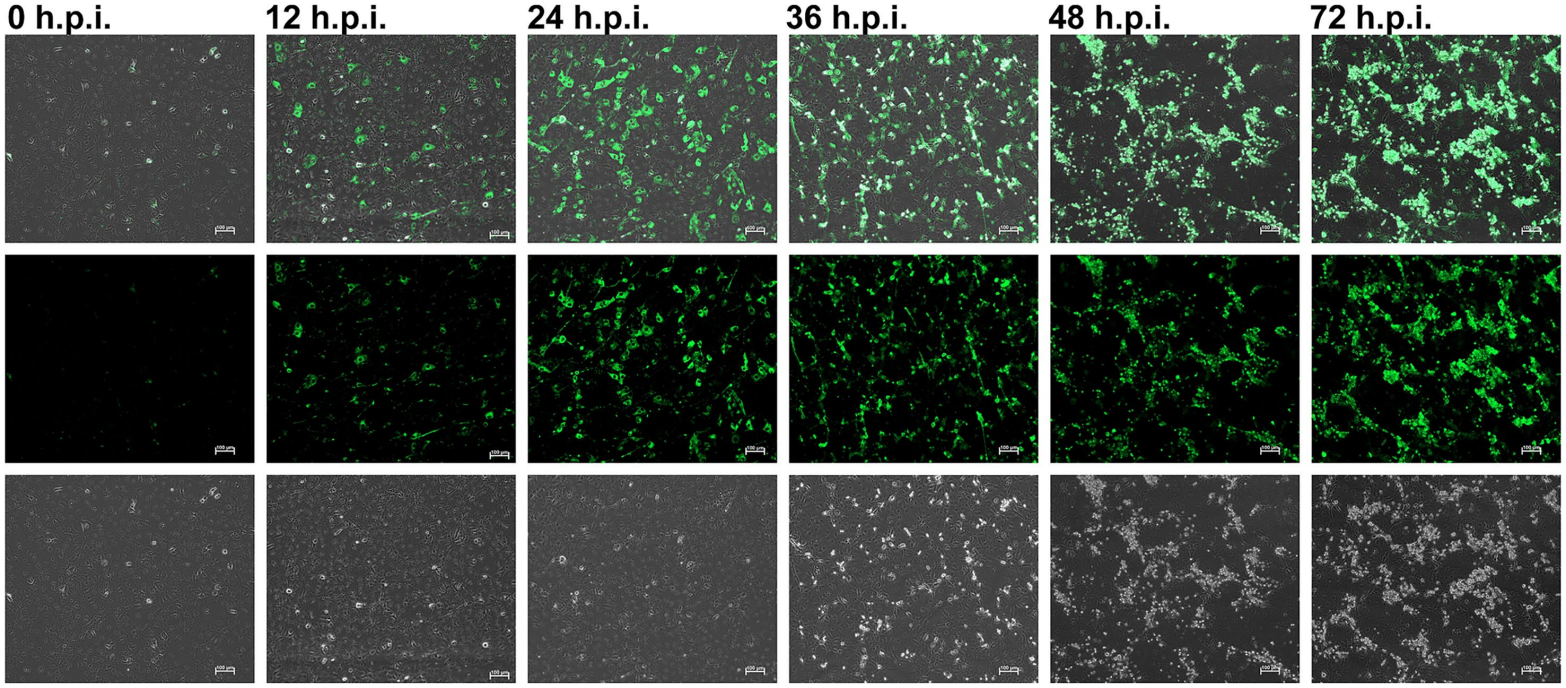
Porcine reproductive and respiratory syndrome virus (PRRSV) infections at a multiplicity of infection (MOI) of 10 were assessed using positive sera at 0, 12, 24, 36, 48, and 72 h post-infection (h.p.i.) through immunofluorescence assay (Scale bar = 100 μm). Exposure and contrast settings were consistent across all images for comparability.

Indirect immunofluorescence assay (IFA) was employed to monitor the temporal dynamics of PRRSV replication in MARC-145 cells (Figure 1). Viral protein expression was visibly detected at 12 h.p.i. The fluorescence intensity and the number of positive cells increased progressively, reaching widespread distribution and saturation between 48 and 72 h.p.i. This exponential increase in viral protein expression directly correlates with the peak transcriptional remodeling observed in the RNA-seq data at 48 h.p.i. Concurrently, significant cytopathic effects (CPE), characterized by cell rounding and detachment (Phase-Contrast panels), became prominent from 36 h.p.i. onwards and intensified markedly by 72 h.p.i. These results confirm the successful establishment of a productive, time-dependent PRRSV infection model for subsequent transcriptomic analysis.

### 3.2 Transcriptional landscapes reveal PRRSV-driven distinct host gene transcription at 12, 24, 36, 48, 72 h.p.i.

**Figure 2:**
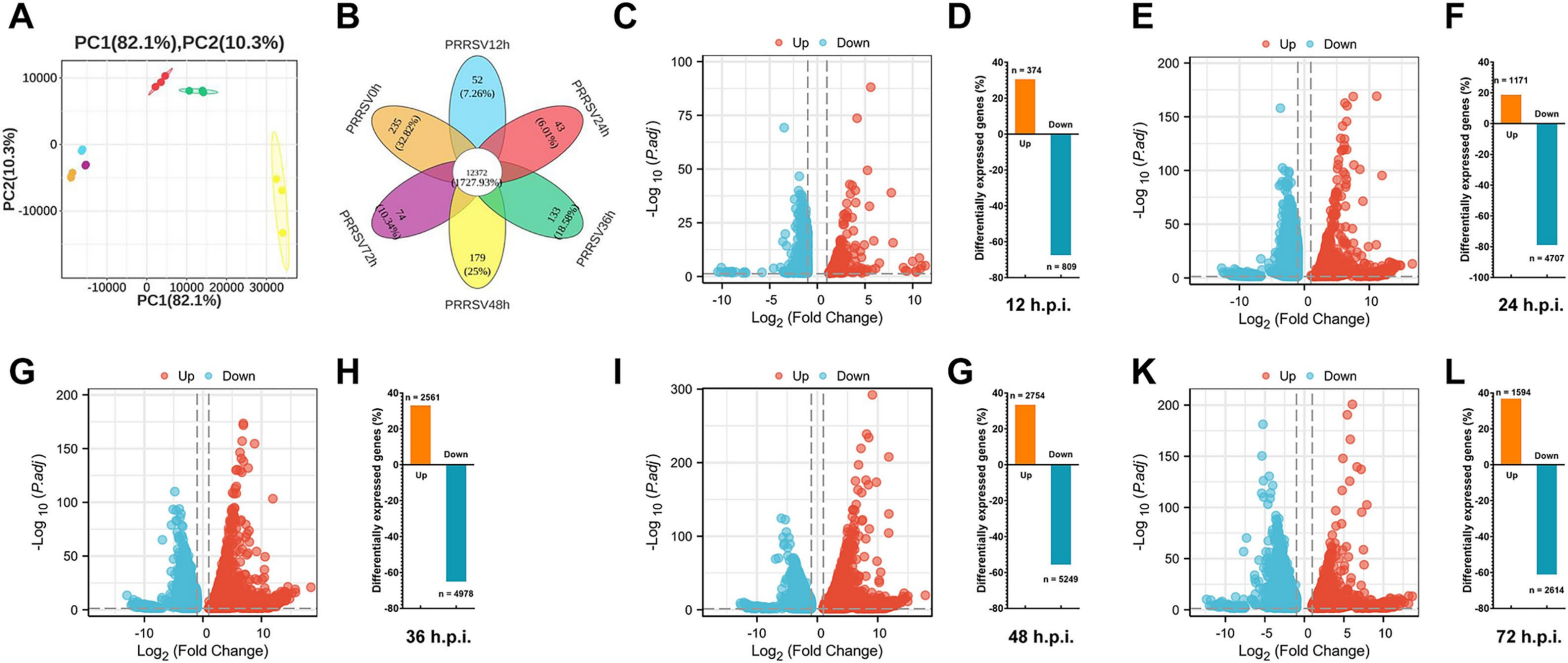
Transcriptomic remodeling and differential gene expression (DEG) in PRRSV-infected MARC-145 cells. **(A)** Principal component analysis (PCA) was conducted. **(B)** Venn diagrams illustrate the overlap of PRRSV-induced differentially expressed genes (DEGs) across 0, 12, 24, 36, 48, and 72 h.p.i., as well as the integrated data from these time points. **(C,E,G,I,K)** Volcano plots depict the fold changes and adjusted *p* values for genes differentially expressed between unstimulated (mock) and PRRSV-stimulated cells. Differentially Expressed Genes (DEGs) are highlighted in red (upregulated) or blue (downregulated) based on the thresholds of Adjusted *p* value (FDR) < 0.05. **(D,F,H,J,L)** The number of upregulated, downregulated, and total DEGs between unstimulated (mock) and PRRSV-stimulated MARC-145 is presented in the transcriptomic data at 0, 12, 24, 36, 48, and 72 h.p.i., respectively.

Principal component analysis (PCA) of the transcriptomic data elucidated a distinct trajectory corresponding to infection time. The tight clustering of biological replicates at each time point (Figure 2A) validates the low sample dispersion and high reproducibility across the time course. Replicates at each time point exhibited tight clustering, while the centroids demonstrated a monotonic progression from 0 h through 12, 24, 36, 48, to 72 h along the principal component. This pattern indicates a progressive and ordered remodeling of the transcriptome, as opposed to stochastic drift. The 0-h cohort was distinctly separated from all post-infection groups, indicating rapid early transcriptional reprogramming that continued to intensify up to 72 h (Figure 2A). Venn diagram analysis of differentially expressed genes (DEGs) in PRRSV-infected MARC-145 cells identified a substantial shared core of 12,372 genes present at all time points, alongside modest time-specific signatures: 0 h, 235 genes (32.8%); 12 h, 52 genes (7.3%); 24 h, 43 genes (6.0%); 36 h, 133 genes (18.6%); 48 h, 179 genes (25.0%); and 72 h, 74 genes (10.3%). The smallest unique gene sets were observed at 12 and 24 h, whereas unique transcription peaked at 48 h and declined by 72 h, suggesting a delayed yet pronounced host response during mid-to-late infection, followed by partial resolution (Figure 2B).

Time-resolved RNA sequencing of MARC-145 cells infected with PRRSV demonstrated extensive transcriptional remodeling, predominantly characterized by downregulation. At 12 h post-infection (h.p.i.), 1,183 differentially expressed genes (DEGs) were identified, with 374 upregulated and 809 downregulated, meeting the specified adjusted *p*-value and log2-fold-change criteria. The extent of transcriptional regulation increased at 24 h.p.i. (1,171 upregulated and 4,707 downregulated) and 36 h.p.i. (2,561 upregulated and 4,978 downregulated), reaching a peak at 48 h.p.i. with 8,003 DEGs, of which 2,754 were upregulated and 5,249 were downregulated, exhibiting larger effect sizes and greater statistical significance. By 72 h.p.i., although widespread transcriptional changes persisted with 4,208 DEGs (1,594 upregulated and 2,614 downregulated), the magnitude of these changes was reduced. Throughout all time points, downregulated genes predominated, suggesting an initial phase of transcriptional suppression followed by a sustained, large-scale reprogramming during the course of PRRSV replication (Figures 2C–L).

### 3.3 Temporal transcriptomic remodeling reveals interconnected immune, metabolic, and pathological reprogramming

**Figure 3:**
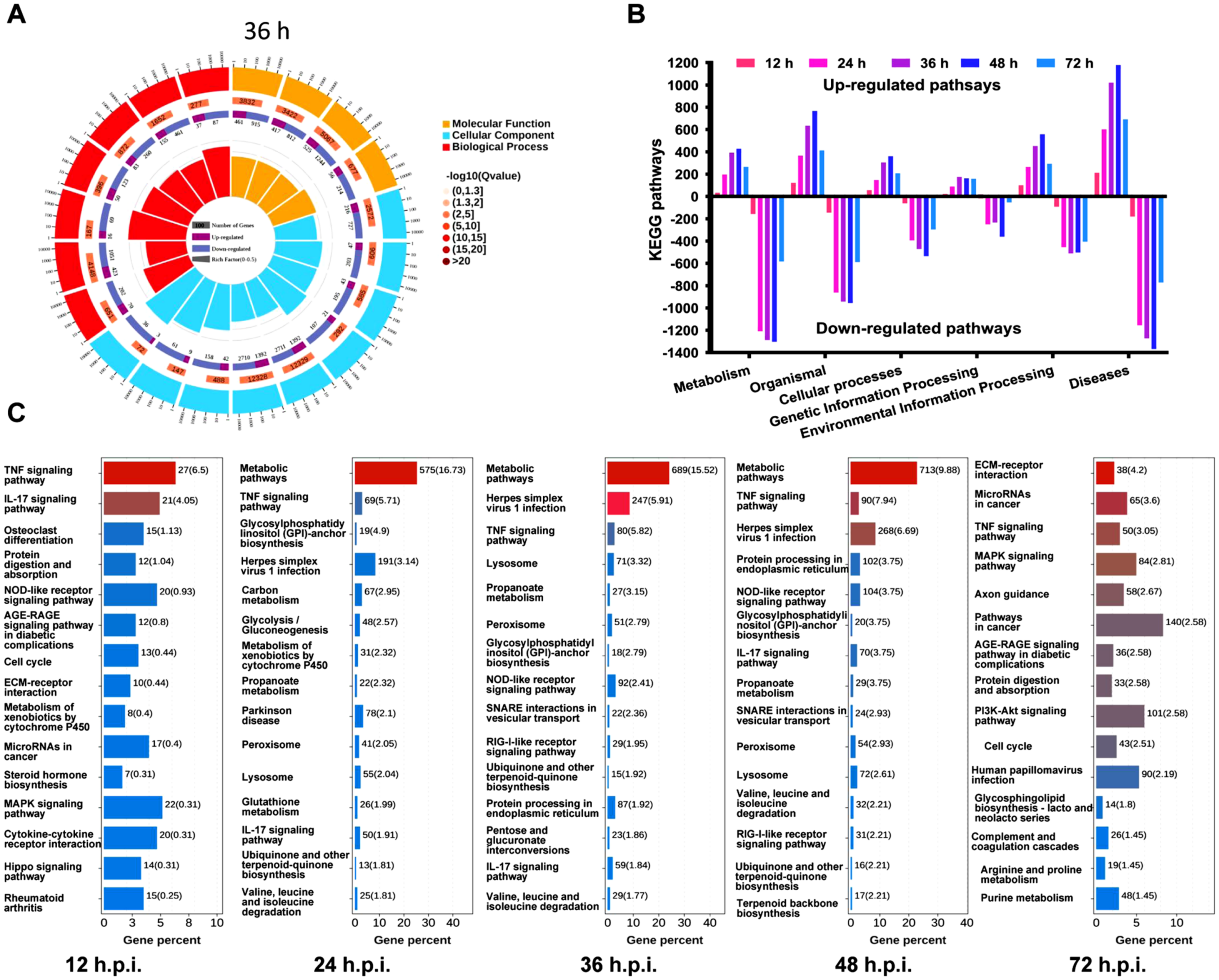
Functional enrichment analysis of DEGs. **(A)** GO analysis of genes exhibiting expression changes at 36 h.p.i., along with integrated data across three time points. **(B)** KEGG analysis of genes with expression changes at 0, 12, 24, 36, 48, and 72 h.p.i., including integrated data. DEGs were categorized based on their involvement in metabolic processes, organismal systems, cellular processes, genetic information processing, environmental information processing, and disease pathways. **(C)** KEGG enrichment analysis focusing on metabolic and immune pathways.

Gene Ontology (GO) enrichment of DEGs revealed a BP-focused response. Up regulated genes were significantly associated with antiviral and immune responses, cytokine signaling, type I interferon response, inflammation, and regulation of apoptosis and autophagy, with high significance (−log_10_Q often > 20) and rich factors near 0.5. Down-regulated genes were linked to cell-cycle progression, RNA processing and translation, and metabolism. CC terms highlighted membranes, lysosome and endosome, and mitochondria, while MF terms focused on cytokine and chemokine receptor binding and hydrolase and oxidoreductase activities (Figure 3A). The Kyoto Encyclopedia of Genes and Genomes (KEGG) pathway enrichment analysis indicated temporal dynamics in pathway regulation. Metabolic and cellular processes were mainly down-regulated, with the most pronounced suppression occurring between 12 and 24 h, which then diminished by 72 h. Genetic and environmental information processing pathways exhibited moderate down-regulation throughout the observed period. In contrast, pathways related to disease showed progressive up-regulation, reaching a peak at 72 h with over 1,000 enriched entries (Figure 3B).

KEGG pathway enrichment analysis of differentially expressed genes in PRRSV-infected MARC-145 cells revealed temporal dynamics in regulated cell death responses. At 12 h.p.i., inflammatory pathways such as TNF signaling, IL-17 signaling, and NOD-like receptor signaling predominated, alongside metabolic shifts in xenobiotic metabolism and cell cycle regulation. By 24 and 36 h.p.i., enrichments shifted toward metabolic reprogramming, including propanoate metabolism, valine and leucine degradation, glycolysis, and peroxisome and lysosome functions, with persistent herpes simplex virus-related and RIG-I-like receptor signaling. Late stages (48 and 72 h.p.i.) featured MAPK, PI3K-Akt, ECM-receptor interaction, and cancer pathways, indicating progressive cellular remodeling and cross-talk between inflammation, metabolism, and cell death machinery (Figure 3C).

### 3.4 Transcriptional signatures of classical regulated cell death pathways exhibit dynamic temporal staging during PRRSV infection

**Figure 4:**
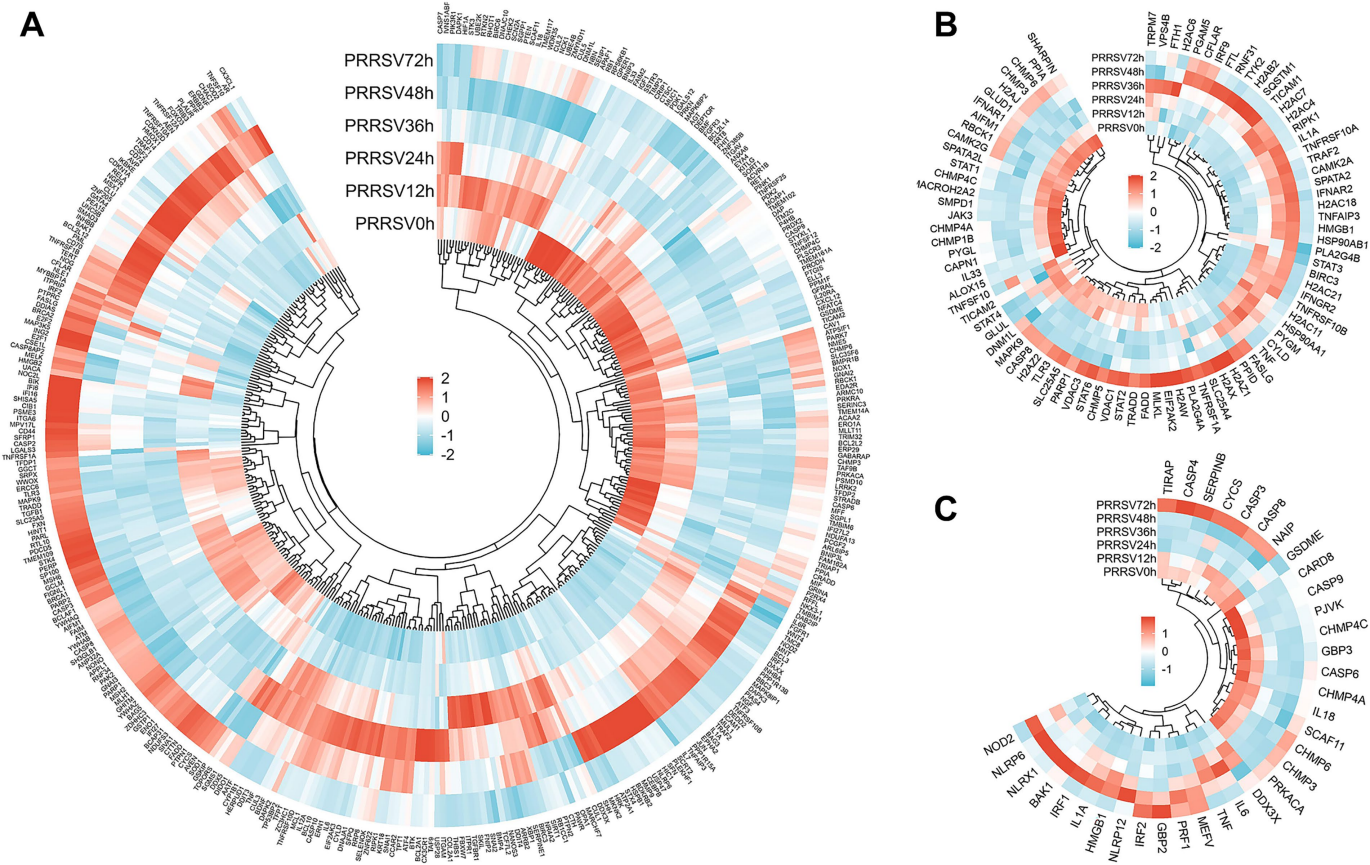
Analysis of DEGs in MARC-145 cells following *in vitro* stimulation with PRRSV reveals temporally coordinated transcriptional modulation of apoptosis, pyroptosis, and necroptosis pathways, with distinct phase-specific activation patterns. Panels **(A–C)** display heatmap analyses of DEGs related to apoptosis (peaking at 48 h.p.i. via ER stress pathways), pyroptosis (early priming with late execution suppression), and necroptosis (sustained transcriptional inhibition throughout infection) in response to PRRSV infection. Functional pathway activation requires validation beyond transcriptional signatures.

Transcriptomic profiling of MARC-145 cells demonstrated a progressive and temporal activation of the apoptosis pathway following PRRSV infection, with peak expression levels observed at 48 h.p.i. Differential expression analysis revealed a significant induction of stress-responsive and pro-apoptotic genes. Notably, the expression of DDIT3 (CHOP) and PPP1R15A, key mediators of endoplasmic reticulum (ER) stress-induced apoptosis, was markedly upregulated, with a log2 fold change (LFC) greater than 6.2. Additionally, transcription factors JUN and ATF3 exhibited an LFC greater than 9.1. This substantial transcriptional remodeling suggests that PRRSV predominantly induces apoptosis through the ER stress-mediated intrinsic pathway. However, the concurrent, pronounced induction of the anti-apoptotic regulator TNFAIP3 (LFC > 9.4) indicates a simultaneous counter-regulatory mechanism, potentially employed by the host or virus, to modulate the extent of cellular demise (Figure 4A).

The time-course RNA-seq analysis of PRRSV-infected MARC-145 cells demonstrated a biphasic modulation of pyroptosis. The early phase was characterized by significant priming, with notable induction of NOD2 and IRF1 (log2 fold change reaching approximately 6 at 48 h) and upregulation of GBP2, resulting in positive net pyroptosis scores between 12 and 36 h, peaking at 48 h (+2.79). Conversely, transcriptional repression was observed in the execution modules, with consistent reductions in CASP4 and GSDME expression (approximately −1.3 and −2.4, respectively), a decline in IL18 levels between 36 and 48 h, and a decrease in CASP3 expression at 48 h (−1.38) before a recovery at 72 h. Consequently, pyroptosis transitioned from transient activation to suppression by 72 h (−0.26). These findings, in conjunction with pathway-level analyses, suggest that PRRSV infection enhances inflammasome priming while inhibiting gasdermin-dependent pore formation and cytokine maturation, thereby delaying terminal pyroptosis while maintaining a pro-inflammatory environment (Figure 4B).

RNA sequencing of MARC-145 cells infected with PRRSV revealed a consistent transcriptional suppression of necroptosis throughout the course of infection. Despite robust upstream signaling—evidenced by significant increases in TNF (log2FC ≈ +5–6), TICAM1 and TRIF (+2.7–4.4), RIPK1 (~ + 3 at 36 and 48 h), and CYLD–SPATA2 (+2 to 3)—the execution of necroptosis was notably inhibited. Specifically, TLR3 expression decreased (−2.1 to −2.3 at 24–48 h), MLKL expression exhibited only a modest and delayed increase (+1.5 at 72 h), and CASP8 levels declined at 24–48 h (−1.1 to −1.25). Potent anti-necroptotic mechanisms were predominant, with significant induction of TNFAIP3 and A20 and BIRC3 and cIAP2 (~ + 6), along with upregulation of TRAF2 and CFLAR. The net necroptosis scores were negative at all measured time points (−0.17, −0.79, −0.37, −0.43, −0.55 from 12 to 72 h). Necroptosis remained transcriptionally constrained, despite the activation of upstream factors TNF and TRIF and RIPK1, which coincided with the induction of A20 and cIAP2, suggesting that ubiquitin-editing mechanisms inhibit necrosome assembly (Figure 4C).

### 3.5 Metal ion and oxidative stress-dependent cell death mechanisms are engaged during PRRSV infection

**Figure 5:**
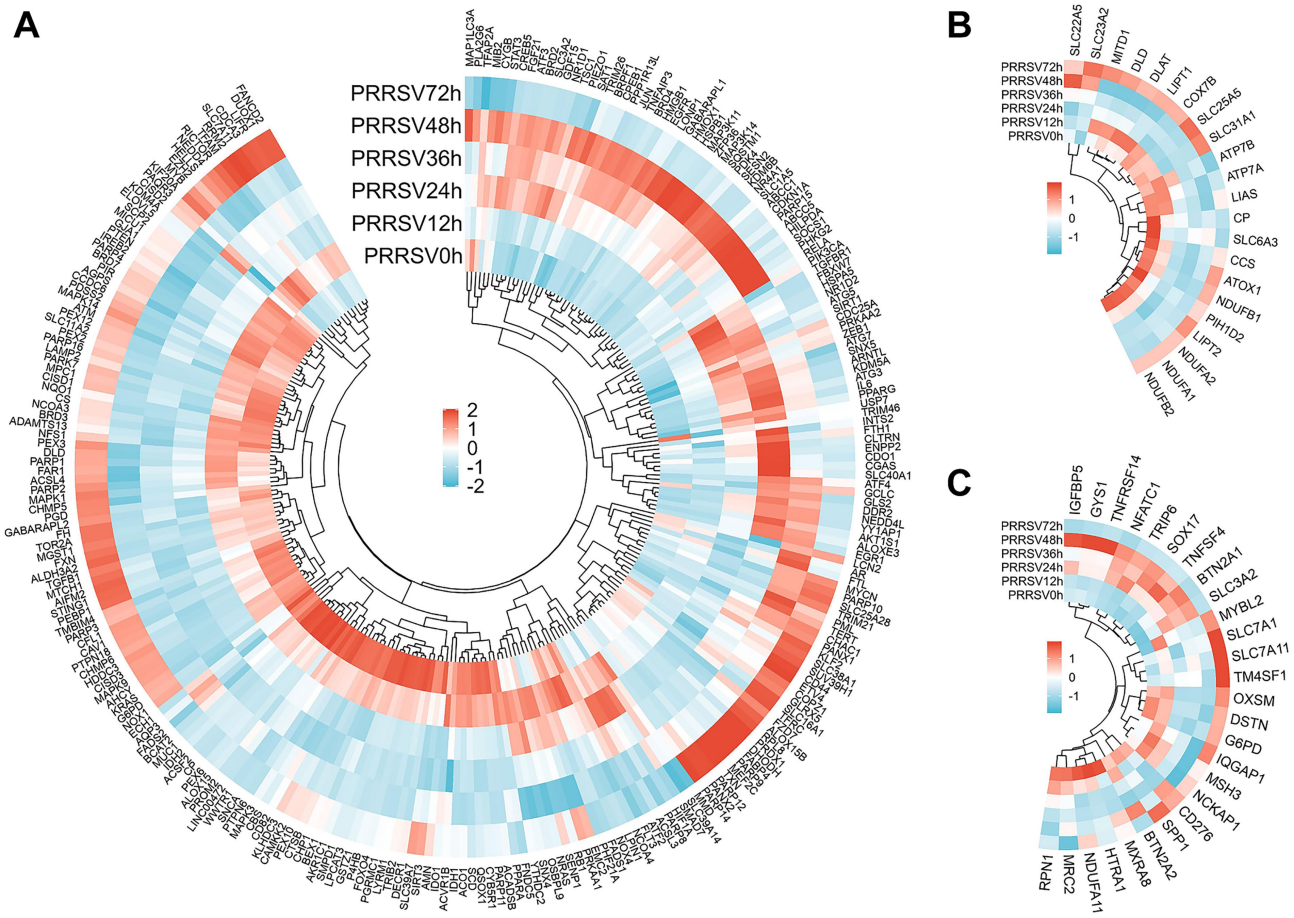
DEGs in MARC-145 cells post-PRRSV stimulation reveal temporally distinct transcriptional regulation of metal ion-dependent cell death pathways: ferroptosis (sustained suppression through 48 h.p.i.), cuproptosis (biphasic pattern with late activation at 72 h.p.i.), and disulfidptosis (mid-phase peak at 36 h.p.i.). Panels **(A–C)** display heatmap analyses of DEGs related to these pathways, illustrating sequential rather than simultaneous engagement. These transcriptional profiles suggest differential temporal windows of pathway susceptibility pending functional validation.

During PRRSV infection, mechanisms of cell death dependent on metal ions and oxidative stress are activated. Transcriptomic profiling revealed the involvement of 254, 23, and 27 genes in the pathways of ferroptosis, cuproptosis, and disulfidoptosis, respectively (Figures 5A–C). These modalities of cell death, regulated by metal ions, demonstrated distinct temporal activation patterns. Transcriptomic analysis of PRRSV-infected MARC-145 cells revealed a predominant transcriptional suppression of ferroptosis. Key modules involved in lipid peroxidation, such as ACSL4, LPCAT3, ALOX15 and ALOX15B, NOX1, and NCOA4, were downregulated between 24 and 48 h, while cytoprotective elements, including HSPB1, SLC40A1, HSPA5, GCLC, and the late induction of SLC7A11, were upregulated. Although pro-ferroptotic genes HMOX1 and ALOXE3 increased between 48 and 72 h, they did not override the anti-ferroptotic program. Pathway scoring, calculated as the mean of pro-ferroptotic minus anti-ferroptotic gene expression, was negative from 12 to 48 h, reaching its lowest point at 36 h, and approached neutrality by 72 h.

Time-course RNA sequencing analysis revealed a biphasic regulation of cuproptosis in PRRSV-infected MARC-145 cells. Initially, pathway scores were negative between 12 and 36 h (ranging from −0.09 to −0.50), which coincided with the downregulation of the lipoylated pyruvate-dehydrogenase module and genes involved in copper uptake and chaperoning (SLC31A1, ATOX1, CCS). From 48 h onward, the pathway exhibited a shift toward activation (net score of +0.20), becoming more pronounced at 72 h (net score of +1.10). This shift was driven by the sustained repression of copper-export pumps (ATP7A at −2.47; ATP7B at −0.97) alongside a partial rebound of SLC31A1 and pyruvate dehydrogenase complex components. Collectively, these dynamics suggest an initial suppression of the copper–lipoylation axis, followed by late intracellular copper accumulation and restoration of lipoylated targets. This combination is predicted to enhance copper binding to mitochondrial lipoylated enzymes, promote proteotoxic aggregation and instability of Fe–S clusters, thereby activating cuproptosis in the later stages of infection.

The results of RNA sequencing has demonstrated a robust yet transient activation of disulfidptosis in MARC-145 cells infected with PRRSV. Pathway scoring, represented by the pro–anti mean log2 fold change, was consistently positive throughout the infection stages, increasing from +1.05 at 12 h to a peak of +2.37 at 36 h. This elevated level persisted at 48 h (+1.68) and decreased by 72 h (+0.95). The observed response was primarily driven by the sustained induction of the cystine antiporter components SLC3A2 and SLC7A11, which exhibited increases of approximately +2.5 and +1.06, respectively. In contrast, G6PD, the main source of NADPH, was downregulated between 24 and 48 h, with fold changes ranging from −1.27 to −1.56. Additionally, NCKAP1 levels decreased at 48 h (−1.03), aligning with the destabilization of the WAVE–actin complex. Collectively, these findings suggest that enhanced cystine influx, coupled with insufficient NADPH regeneration, leads to intracellular disulfide accumulation and cytoskeletal crosslinking, key features of disulfidptosis, during the mid-infection phase, followed by a partial recovery in the later stages.

### 3.6 Transcriptional signatures of organellar stress-driven death pathways demonstrate temporal coordination

**Figure 6:**
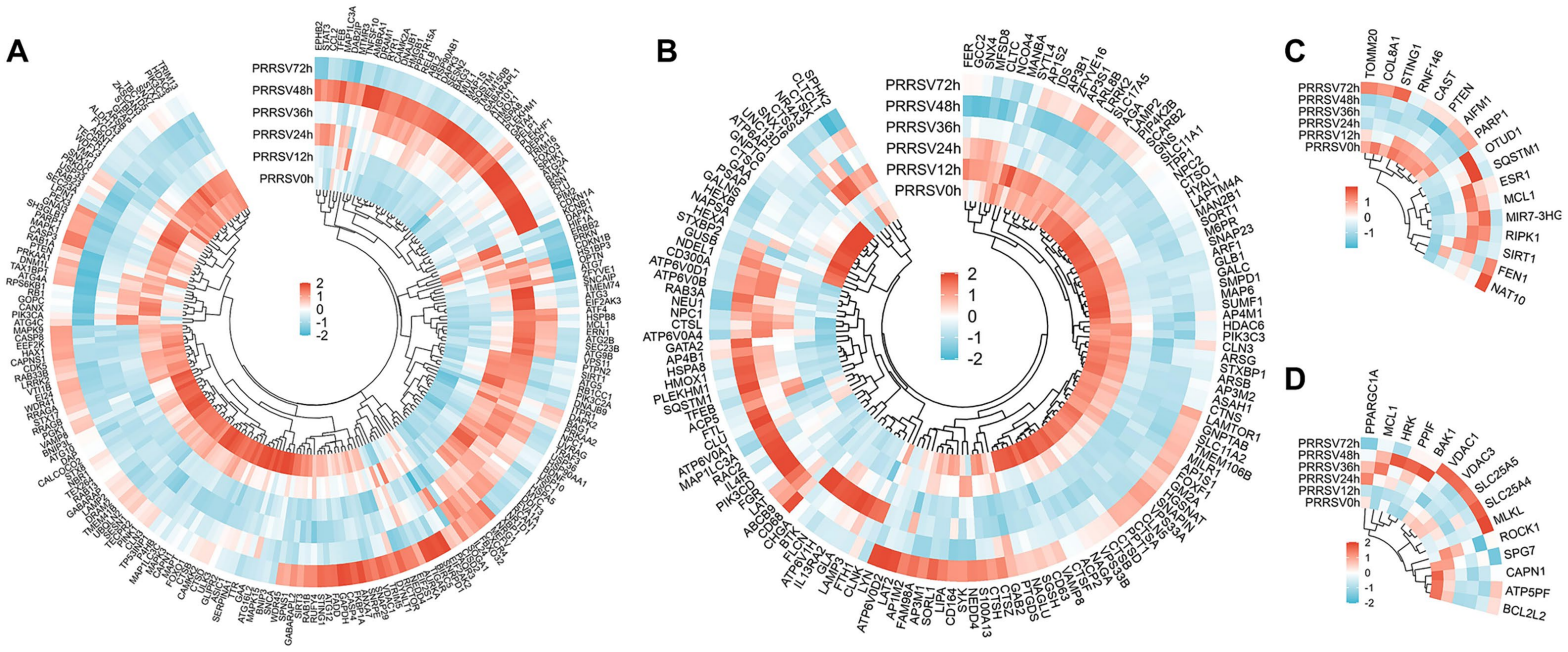
DEGs in MARC-145 cells after PRRSV-specific stimulation demonstrate temporally orchestrated transcriptional remodeling of organellar stress-driven death pathways with distinct phase-specific patterns: autophagy shows flux-impaired phenotype peaking at 24–48 h.p.i., lysosome-dependent cell death exhibits early transient inclination followed by sustained suppression, paraptosis progressively activates through IGF1R-UPR signaling peaking at 48 h.p.i., and MPT-driven necrosis displays biphasic regulation with late sensitization at 72 h.p.i. Panels **(A–D)** illustrate heatmap analyses of DEGs associated with these pathways. These coordinated transcriptional programs suggest temporally staged organellar dysfunction rather than simultaneous pathway execution, requiring functional validation of pathway activity states.

The differential expression analysis identified 198, 134, 25, and 15 genes associated with autophagy, lysosome-dependent cell death, paraptosis, and MPT-driven necrosis pathways, respectively (Figures 6A–D). These organelle-associated death mechanisms exhibited distinct yet interconnected temporal patterns. A time-course RNA-seq analysis of PRRSV-infected MARC-145 cells demonstrated dynamic remodeling of autophagy. Pathway scoring revealed mild repression at 12 h (−0.27), activation at 24 and 48 h (peaking at +1.06 at 48 h), and a return to near-baseline levels by 72 h (+0.07). The initiation and transcriptional modules, including RB1CC1 and FIP200, ATG3 and 5 and 7, TFEB, and UVRAG, were induced, whereas the maturation and fusion machinery, such as PIK3C3 and VPS34, STX17–SNAP29–VAMP8, and LAMP2, were attenuated, with a decrease in GABARAP and a transient increase in GABARAPL1 at 48 h. Notably, SQSTM1 and p62 accumulated significantly at 24–48 h. Collectively, these transcriptional findings support an initiation-heavy, autophagy phenotype, wherein PRRSV facilitates autophagosome biogenesis through TFEB and ATG programs while inhibiting VPS34 SNARE-dependent autophagosome–lysosome fusion. This inhibition limits cargo clearance and potentially provides membranes and metabolites that favor viral replication.

RNA sequencing analysis revealed an initial, transient inclination toward lysosomal cell death in PRRSV-infected MARC-145 cells, which was followed by a sustained repression phase. Pathway scores were recorded as +0.24 at 12 h and +0.11 at 24 h, before transitioning to negative values at 36 to 72 h (−0.77, −0.25, −0.40). The early inclination was characterized by a significant induction of CTSS (log2FC approximately +3.1 at 24 h and +2.2 at 48 h), accompanied by a moderate upregulation of CTSL. Subsequently, key executors were downregulated, including SMPD1 (−2.66 at 36 h), CTSB, CTSD, and CTSA (approximately −1.7), as well as LAMP2 (ranging from −1.07 to −1.42 between 24 and 48 h). Concurrently, ATP6V0D1 expression increased (approximately +1.09 at 36 h), indicating enhanced lysosomal acidification. Collectively, these findings suggest that early signaling prone to lysosomal membrane permeabilization (LMP) is attenuated during the mid-to-late stages of infection through transcriptional suppression of acid sphingomyelinase and key cathepsins, alongside alterations in lysosomal homeostasis, thereby restricting the execution of lysosome-dependent cell death.

The temporal transcriptomic analysis demonstrated a significant activation of paraptosis in MARC-145 cells infected with PRRSV. The net pathway scores, calculated as the difference between pro- and anti-paraptotic mean log2 fold changes, were +0.27 at 12 h, +0.93 at 24 h, +0.91 at 36 h, reaching a peak of +0.98 at 48 h, and subsequently decreasing to +0.12 at 72 h. Key regulatory elements were markedly upregulated, including IGF1R (ranging from +2.07 to +2.98 to +2.80 between 24 and 48 h), the PERK–eIF2α–ATF4–DDIT3 pathway (with DDIT3 approximately +6.0 at 24 to 48 h and ATF4 at +1.27 and +1.14), XBP1 (ranging from +1.10 to +1.34), EIF2S1, BAG3 (increasing from +3.55 to +4.54), and HSPA4 (rising from +1.67 to +2.20). Concurrently, there was a reduction in PSMD14 (−0.48 at 48 h) and a late decrease in PDCD6IP and ALIX, aligning with proteostasis stress and endoplasmic reticulum dilation, while CASP3 levels declined (−1.38 at 48 h), indicating a non-caspase-dependent cell death pathway. Overall, PRRSV infection induces an IGF1R-UPR and ISR–chaperone signature that facilitates endoplasmic reticulum and mitochondrial vacuolization and paraptotic cell death, with partial recovery observed at 72 h, likely mediated by PPP1R15A and GADD34 feedback mechanisms.

Time-resolved RNA sequencing of PRRSV-infected MARC-145 cells demonstrated a biphasic regulation of MPT-driven necrosis. Analysis of pathway scores (pro–anti mean log2 fold change) revealed a mild activation at 12 h (+0.31), followed by suppression at 24 to 48 h (−0.41, −0.36, −0.17), and a renewed activation at 72 h (+0.61). Mechanistically, PPIF and CypD expression was significantly induced between 24 and 72 h, peaking at +3.39, whereas ATP5PF and VDAC1 and 3 levels decreased at 24 to 48 h (to −2.24 and approximately −1.1 and −1.5, respectively), coinciding with the upregulation of MCL1 (to +1.27), collectively restraining MPT mid-course. During the late phase of infection, there was a rebound in VDAC expression, induction of BAK1 (+1.16 at 48 h), and a significant reduction in PPARGC1A at 72 h (−1.63), which reduced mitochondrial resiliency. MPT-driven necrosis exhibited a biphasic pattern, with mid-course restraint (ATP5PF and VDAC downregulation, MCL1 upregulation) followed by late sensitization (CypD upregulation, VDAC rebound, PPARGC1A downregulation) However, sustained elevation of CypD, along with compromised oxidative phosphorylation and biogenesis defenses, facilitates late MPT sensitization and the execution of necrosis.

### 3.7 DNA damage response and pH regulation pathways show coordinated transcriptional activity during PRRSV-induced cell death

**Figure 7:**
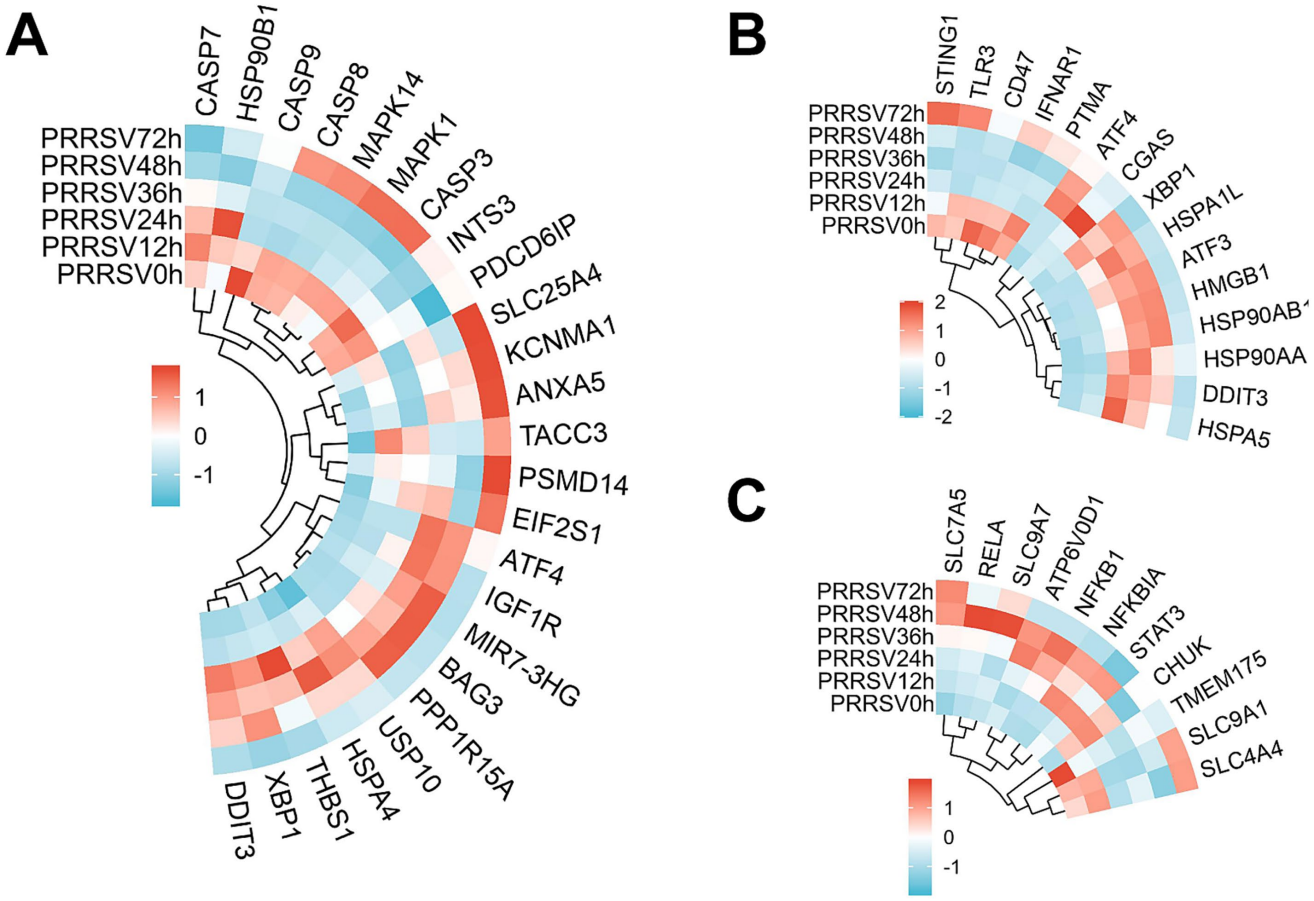
Differentially expressed genes (DEGs) in MARC-145 cells following in vitro stimulation with PRRSV reveal temporally divergent transcriptional regulation of DNA damage-responsive and pH-dependent death pathways: parthanatos shows sustained transcriptional suppression throughout infection (net scores negative at all time points), alkaliptosis exhibits progressive activation peaking at 72 h.p.i., while immunogenic cell death (ICD) signatures remain persistently elevated from 12 h.p.i. onward. Panels **(A–C)** present heatmap analyses of DEGs associated with parthanatos, alkaliptosis, and ICD at sequential time points post-PRRSV infection. These contrasting temporal patterns suggest phase-specific vulnerabilities and stress responses inferred from transcriptional programs pending functional validation.

The Parthanatos and alkaliptosis pathways, which regulate DNA metabolism-dependent and pH homeostasis-dependent cell death, respectively, exhibited coordinated temporal activation. Differential expression analysis identified 17 genes associated with Parthanatos and 11 genes related to alkaliptosis (Figures 7A,B). A time-course RNA-seq analysis of PRRSV-infected MARC-145 cells revealed sustained repression of the Parthanatos pathway. The pathway net scores (pro–anti mean log2FC) were −0.13 at 12 h, −1.11 at 24 h, −0.97 at 36 h, reaching a nadir of −1.41 at 48 h, and −0.26 at 72 h. Despite an increase in RIPK1 (~ + 3 at 36–48 h) and FEN1, the core execution nodes were attenuated: PARP1 (~ − 0.97) and AIFM1 (~ − 1.52) decreased, and STING1 and TOMM20 were reduced between 24 and 48 h. Concurrently, anti-Parthanatos mechanisms were enhanced, as evidenced by the upregulation of SIRT1 (+1.0–1.8 at 12–36 h), SQSTM1 and p62 (+3.1–4.9 at 24–48 h), OTUD1, and MCL1 and NAT10, which counteracted declines in RNF146 and CAST. Collectively, PRRSV attenuates the PARP1 → PAR accumulation→AIFM1 nuclear translocation cascade and mitochondrial–STING signaling, while reinforcing deacetylation and autophagic buffering, thereby suppressing Parthanatos execution with partial late relief.

The time-course RNA sequencing analysis demonstrated a progressive activation of alkaliptosis during PRRSV infection in MARC-145 cells. The net pathway scores, calculated as the mean log₂ fold change of pro- versus anti-alkaliptosis factors, were positive at all observed time points: +0.41 at 12 h, +0.34 at 24 h, +0.51 at 36 h, +0.34 at 48 h, and reaching a peak of +0.77 at 72 h. Key components of the NF-κB pathway were upregulated, with NFKB1 showing an increase up to +4.05 and RELA and CHUK exhibiting moderate elevation. Concurrently, there was a sustained increase in the amino acid transporter SLC7A5, ranging from +1.43 to +2.84. Counter-regulatory elements displayed varied responses: NFKBIA significantly increased (+5.11 to +4.93 between 24 and 48 h), SLC9A1 showed a transient decrease (−1.10 to −0.70), while the lysosomal channel TMEM175 was consistently downregulated (−2.04 to −3.10, maintaining −2.01 at 72 h), and ATP6V0D1 exhibited a mid-course increase (+1.09). Overall, the transcriptional activity driven by NF-κB and the enhanced nutrient influx, in conjunction with disrupted lysosomal pH and ion homeostasis and temporary suppression of Na^+^ and H^+^ exchange, promote intracellular alkalinization, culminating in a state prone to late-phase alkaliptosis.

### 3.8 Immunogenic cell death signatures emerge during PRRSV infection

A temporal RNA sequencing analysis of PRRSV-infected MARC-145 cells demonstrated a sustained activation of immunogenic cell death (ICD). The net pathway scores, calculated as the mean log₂ fold change of pro- versus anti-ICD factors, remained strongly positive across all examined time points: +1.98 at 12 h, +1.92 at 24 h, +2.25 at 36 h, +2.23 at 48 h, and +2.23 at 72 h. This signature was predominantly characterized by a substantial induction of HMGB1 (approximately +6.0 from 12 to 72 h), indicative of damage-associated molecular pattern (DAMP) release, and the activation of endoplasmic reticulum stress and unfolded protein response (UPR) signaling via XBP1, with scores of +1.32, +1.10, and +1.34 at 24 to 48 h. Simultaneously, the repression of the “do not-eat-me” signal CD47 (ranging from −0.48 to −2.08) suggests enhanced phagocytic clearance. Contrastingly, other regulatory nodes exhibited varied responses: IFNAR1 expression decreased to −1.75 at 48 h, potentially moderating type-I interferon amplification, while HSPA5 and BiP expression increased from +2.10 to +1.11 at 24 to 48 h. Collectively, the interplay of DAMP release, UPR engagement, and diminished antiphagocytic signaling underpins the persistent activation of ICD from the mid-infection stage onward (Figure 7C).

### 3.9 Temporal gene expression clustering in PRRSV-infected cells

**Figure 8:**
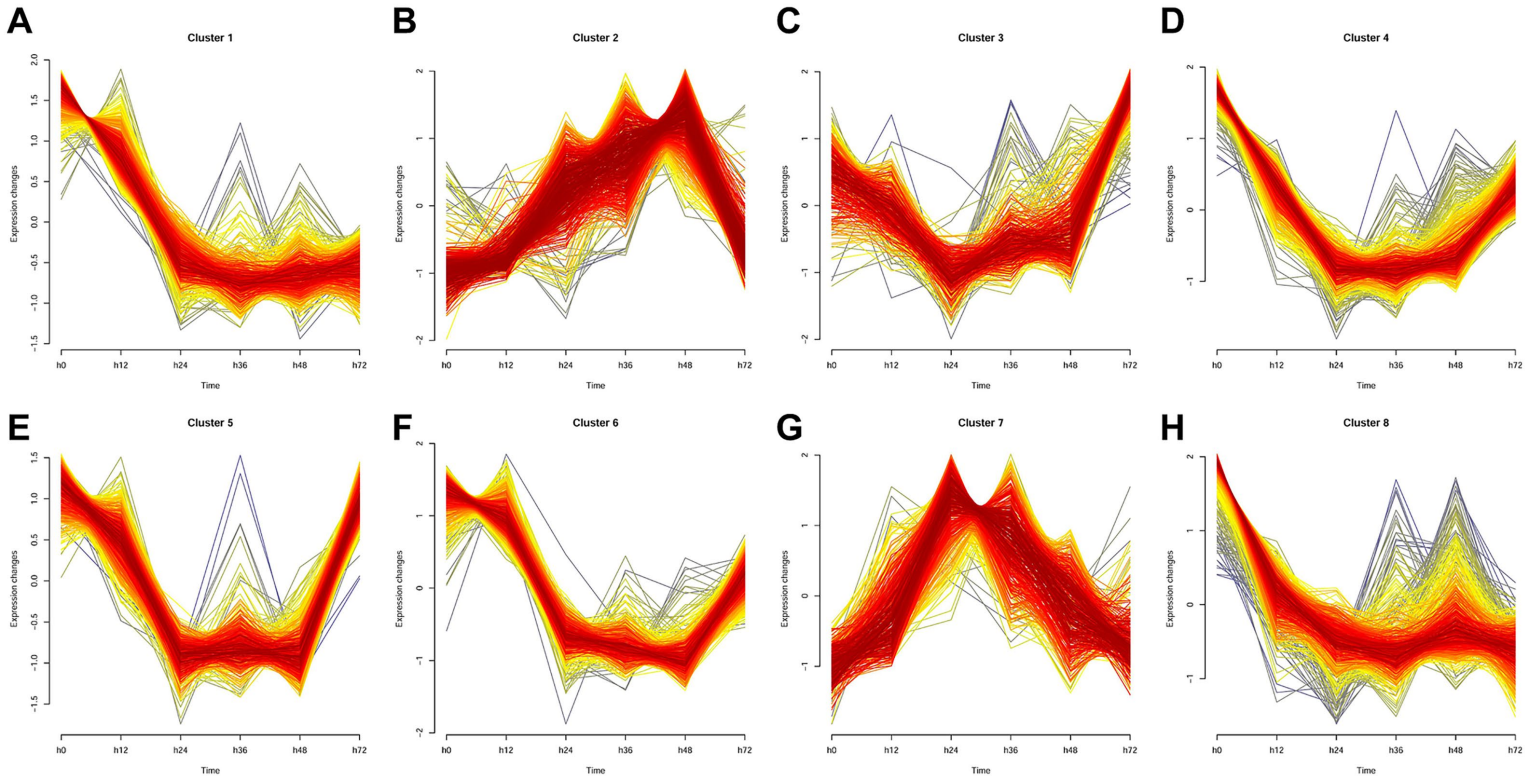
Temporal clustering using the Mfuzz algorithm identified eight distinct transcriptional modules during PRRSV infection. Specifically, Mfuzz analysis partitioned the transcriptomes of PRRSV-infected MARC-145 cells into eight clusters, labeled **(A–H)**.

Mfuzz clustering of transcriptomic data from PRRSV-infected MARC-145 cells revealed eight distinct temporal patterns across 0–72 h.p.i. (Figure 8). Clusters A, D, E, F, and H exhibited early downregulation, likely reflecting suppression of antiviral genes and initial RCD inhibition. Clusters B and G showed mid-phase upregulation, correlating with activation of apoptotic and pyroptotic pathways, including caspase and inflammasome effectors. Cluster C displayed a biphasic pattern, suggesting cross-regulation between necroptosis and ferroptosis machinery. These dynamics underscore PRRSV’s sequential manipulation of RCD subroutines, promoting immune evasion and cytopathology.

### 3.10 Temporal profiling of cell death markers confirms sequential activation of stress-driven programs

**Figure 9:**
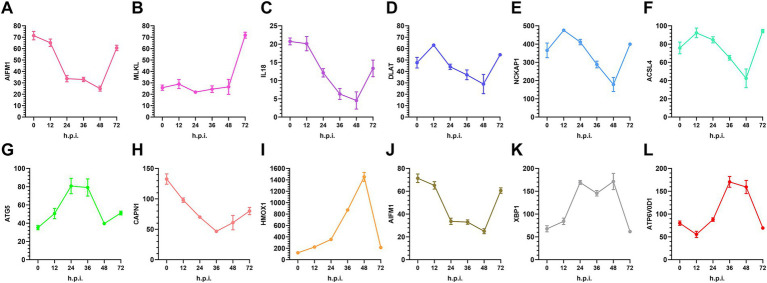
The temporal expression profiles of selected genes associated with regulated cell death, including **(A)** AIFM1, **(B)** MLKL, **(C)** IL18, **(D)** DLAT, **(E)** NCKAP1, **(F)** ACSL4, **(G)** ATG5, **(H)** CAPN1, **(I)** HMOX1, **(J)** AIFM1, **(K)** XBP1, and **(L)** ATP6V0D1, were analyzed in PRRSV-infected MARC-145 cells. Normalized RNA-seq counts for key markers of apoptosis, pyroptosis, necroptosis, ferroptosis, endoplasmic reticulum (ER) stress, autophagy, lysosomal activity, and immunogenic cell death (ICD) were assessed at 0, 12, 24, 36, 48, and 72 h.p.i. The data reveal early inflammatory and stress priming, mid-phase ER-associated activation, and late-stage metabolic and pH-related remodeling. Results are expressed as mean ± standard deviation (SD) from three independent infection experiments.

Transcriptional profiling of key cell death markers validated the temporal progression from suppressed lytic pathways to stress-driven non-apoptotic programs during PRRSV infection (Figure 9). Consistent with constrained necroptosis execution, MLKL expression remained minimal throughout infection, while AIFM1 decreased progressively, confirming parthanatos suppression. The pyroptosis effector IL1B showed continuous downregulation despite inflammatory priming, supporting uncoupled inflammasome activation. Conversely, markers of cellular stress responses exhibited pronounced mid-infection activation. XBP1 showed peak expression at 24 and 36 h.p.i., with similar magnitude at both time points, coinciding with ATG3 upregulation, indicative of ER stress-driven autophagy initiation. The dramatic induction of HMOX1 at 48 h, alongside elevated ATP6V0D1 expression, revealed concurrent oxidative stress and lysosomal acidification attempts. Notably, the ferroptosis-associated factors ACSL4 and NCOA4 showed variable patterns, with NCOA4 declining after initial elevation, consistent with ferroptosis resistance. These temporal expression profiles corroborate the model of PRRSV-orchestrated cell death programming that transitions from early lytic suppression through ER stress-mediated responses to late metabolic vulnerabilities.

## 4 Discussion

Time-resolved transcriptomic analysis of MARC-145 cells infected with PRRSV demonstrated a systematic and monotonic reorganization of the host gene expression program over a period of 0, 12, 24, 36, 48, 72 h.p.i. The data exhibited tight clustering among replicates and a progressive divergence from mock-infected controls, suggesting coordinated, infection-driven dynamics rather than random variation. Differential gene expression reached its peak at 48 h.p.i., characterized predominantly by down-regulation, which aligns with an initial phase of global suppression followed by extensive reprogramming. Gene Ontology (GO) and Kyoto Encyclopedia of Genes and Genomes (KEGG) pathway analyses revealed an early and sustained enrichment of antiviral and type-I interferon (IFN) signaling, as well as cytokine signaling pathways, alongside a concurrent repression of pathways related to the cell cycle, RNA processing, and metabolism. Disease-associated pathways showed a gradual increase, peaking at 72 h.p.i., indicative of cumulative pathophysiological effects.

Classical RCD circuits exhibited distinctive temporal logic characterized by sequential rather than simultaneous engagement. Apoptosis transcriptional programs were progressively induced via ER-stress pathways (DDIT3 and PPP1R15A; JUN and ATF3), reaching peak expression at 48 h.p.i. This ER stress signature was counterbalanced by concurrent upregulation of the anti-apoptotic regulator TNFAIP3, suggesting transcriptional profiles consistent with delayed but not completely blocked apoptotic execution. Pyroptosis demonstrated early inflammasome priming (NOD2, IRF1, GBP2 upregulation at 12–36 h.p.i.) but showed mid-to-late repression of executioner molecules (CASP4, GSDME, IL18 suppression at 36–72 h.p.i.), indicating temporally uncoupled priming and execution phases. Necroptosis remained transcriptionally constrained throughout all time points despite upstream signal induction (TNF, TRIF, RIPK1), coinciding with sustained elevation of inhibitory factors (A20, cIAP2), suggesting that ubiquitin-editing mechanisms prevent necrosome assembly across the infection time course. Collectively, these temporally staged transcriptional programs suggest PRRSV employs phase-specific strategies to sustain pro-inflammatory signaling while suppressing membrane-disruptive lytic death, potentially facilitating viral replication while minimizing catastrophic cell loss. Functional validation of these inferred pathway states through protein-level and activity assays is required to confirm these transcription-based predictions.

Non-classical modalities provide further refinement to this landscape. The ferroptosis-related modules responsible for regulating lipid peroxidation (including ACSL4, LPCAT3, ALOX15, NOX1, and NCOA4) were largely downregulated, whereas cytoprotective elements (such as HSPB1, SLC40A1, HSPA5, GCLC, and later SLC7A11) were upregulated. Pathway scores remained negative up to approximately 48 h.p.i., gradually approaching neutrality by 72 h.p.i., suggesting that viral mechanisms may inhibit iron-dependent lethal lipid damage. Conversely, cuproptosis exhibited a transition from early suppression to late activation, in conjunction with the repression of ATP7A and B and a partial recovery of copper uptake and lipoylated enzyme modules, indicating a potential late-stage susceptibility to copper-lipoylation toxicity. Disulfidptosis reached its peak mid-course, driven by the induction of SLC3A2 and SLC7A11, coupled with a deficiency in NADPH.

Organelle-centric programs displayed coordinated yet uncoupled characteristics (40–42). Autophagy was marked by initiation-heavy but flux-impaired features, with TFEB and ATG induction and VPS34 and SNARE and LAMP2 attenuation leading to SQSTM1 accumulation. This pattern supports membrane and metabolite provision while limiting cargo clearance, benefiting viral biogenesis. Lysosome-dependent cell death showed a brief early increase (CTSS and CTSL upregulation) followed by sustained repression (decreased ASM, major cathepsins, and LAMP2). Paraptosis increased via the IGF1R–PERK and eIF2α–ATF4–DDIT3–XBP1–chaperone pathways, with diminished proteostasis capacity (PSMD14 and late PDCD6IP downregulation). MPT-driven necrosis exhibited a biphasic pattern, with mid-course restraint (ATP5PF and VDAC downregulation, MCL1 upregulation) followed by late sensitization (CypD upregulation, VDAC rebound, PPARGC1A downregulation). These transcriptional findings suggest that PRRSV exploits ER and mitochondrial stress to alter cellular fate while potentially delaying terminal lytic processes until late in the infection.

Parthanatos associated with DNA damage was inhibited (indicated by decreased PARP1 and AIFM1 and increased SIRT1 and SQSTM1, among other factors), whereas alkaliptosis progressively intensified, correlating with NF-κB activity and disruptions in amino acid transport and lysosomal pH and ion balance (43–45). This pattern suggests a heightened vulnerability to pH-dependent cytotoxicity in later stages. Simultaneously, robust signatures of immunogenic cell death were observed, characterized by increased HMGB1, activation of UPR-XBP1, and repression of CD47. These findings, based on sustained transcriptional signatures, imply that PRRSV-induced stress may enhance DAMP signaling and the potential for phagocytic clearance, even in the context of altered interferon signaling.

The repression of DNA-damage-associated parthanatos (characterized by decreased PARP1 and AIFM1 and reinforcement of SIRT1 and SQSTM1, among other factors) was observed, while alkaliptosis progressively increased, correlating with NF-κB activity and disruptions in amino acid transport and lysosomal pH and ion homeostasis (43, 46, 47). This pattern suggests a heightened susceptibility to pH-dependent cytotoxicity in the later stages. Concurrently, robust signatures of immunogenic cell death emerged, indicated by increased HMGB1, activation of the UPR-XBP1 pathway, and repression of CD47. These findings imply that PRRSV-induced stress may amplify DAMP signaling and enhance phagocytic clearance potential, despite modulation of interferon signaling.

These observations align with current understandings of regulated cell death taxonomy and viral immune-evasion strategies, viruses activate inflammatory pathways while modulating ubiquitin signaling, endoplasmic reticulum stress, and organellar quality control to evade premature, membrane-disruptive cell death and to optimize replication environments. Although MARC-145 cells provide a highly tractable and widely used system for dissecting PRRSV host interactions, it is important to acknowledge that they do not fully recapitulate the innate immune landscape of primary porcine alveolar macrophages (PAMs), the principal target cells during natural infection. As epithelial-derived cells, MARC-145 cells exhibit lower basal expression of key pattern-recognition receptors (e.g., TLRs, RIG-I like sensors), inflammasome components, and several executional nodes of RCD pathways compared with PAMs, which are primed for rapid cytokine release, phagocytic signaling, and mitochondrial–lysosomal crosstalk. These lineage-specific differences likely influence both the magnitude and the timing of transcriptional repression or activation observed across apoptotic, necroptotic, ferroptotic, and autophagy-related modules in our dataset. As such, the temporal RCD architecture characterized here should be interpreted as a model-specific transcriptional program reflecting epithelial cell biology rather than a direct mirror of *in vivo* macrophage responses. Nonetheless, the staging patterns revealed in this high-resolution atlas provide testable hypotheses for future validation in PAMs.

## 5 Conclusion

PRRSV induces a staged transcriptional reprogramming characterized by early inflammatory priming with global suppression, mid-course ER-stress-driven apoptosis/paraptosis and flux-impaired autophagy, persistent ICD signaling, and late metabolic/pH vulnerabilities (cuproptosis, alkaliptosis), while actively restraining lytic pathways (necroptosis, parthanatos, and ferroptosis). This architecture explains how the virus balances replication permissiveness with controlled cytopathology and identifies temporal windows for therapeutic manipulation restoring degradative flux, disinhibiting necroptotic execution, or timing copper dependent strategies, while leveraging ICD to augment antiviral immunity.

## Data Availability

The data presented in the study are deposited in the NCBI Short Read Archive (SRA) repository, accession number PRJNA1372434 (https://www.ncbi.nlm.nih.gov/bioproject/1372434). The original contributions presented in the study are included in the article/supplementary material. Further inquiries can be directed to the corresponding author/s.
